# New reflexes during resting motor threshold determinations

**DOI:** 10.1177/00048674231166891

**Published:** 2023-04-12

**Authors:** Saxby Pridmore, Renee Morey, Yvonne Turnier-Shea, Marzena Rybak

**Affiliations:** 1 Discipline of Psychiatry, University of Tasmania, Hobart, TAS, Australia; 2 TMS Unit, Saint Helens Private Hospital, Hobart, TAS, Australia

When a patient is to receive transcranial magnetic stimulation (TMS) it is necessary to first determine the resting motor threshold (RMT)—the minimum intensity of magnetic pulse required to activate the target area. The RMT is measured by placing the coil on the scalp/hair over the motor area and delivering various quantities of energy—we are looking to trigger a slight motor response of a contralateral finger. The precise location of the motor cortex is not initially known and is found by slight relocations of the coil.

During this process we sometimes notice the patient sometimes evidences a twitch in the cheek (on the same side) or a blink of an eye (on the same or both sides).

It has been concluded the twitch in the cheek is a contraction of the masseter muscle and the blinking is contraction of the orbicularis oculi. These contractions are considered benign phenomenon and clinicians in the field dismiss them as the result of ‘superficial nerve stimulation’.

But what we mean by ‘superficial nerve stimulation’ is unclear. Neural tissue is more easily activated than muscle tissue, and nerve depolarization results in activation of all downstream muscle fibers. Thus, the depolarization of ambient nerves could possibly explain these events.

However, examination of ‘superficial nerve stimulation’ as the explanation for these masseter and orbicularis oculi contractions raises questions. When seeking finger movement, we stimulate in a region some centimeters superior to the pinna and a little posterior to the coronal plane through the tragus. Given these landmarks, it is unclear (1) how the masseter nerve could be depolarized, as it is at least 12 cm distant and always deep to the masseter, and (2) why we fail to stimulate the trunk of the facial nerve after exiting the stylomastoid foramen and before it divides into branches—it is closer than the masseter nerve and would produce contractions across the face. The ‘the superficial nerve stimulation’ explanation is lacking.

When the masseter or orbicularis oculi is triggered and the position and the strength of the stimulus are held constant, the contractions remain constant in timing and strength—reminiscent of reflexes. We therefore searched for tissue components which could support the theory that the twitches sometimes observed during RMT determinations are reflex responses—i.e. magnetic pulses causing depolarization of sensory nerves, which lead to activation of motor nerves and muscle contraction.

## A possible mechanism for reflexes

Seeking the motor cortex, we place the center of the coil on the scalp/hair superior to the pinna and posterior to the tragus (our initial estimate of the location of the motor cortex). At this site, two cranial nerve dermatomes abut.

The ophthalmic nerve (first branch of the trigeminal nerve) dermatome serves an anteroposterior strip of scalp running some 5 cm lateral to the midline. The mandibular nerve (third branch of the trigeminal nerve) dermatome forms an adjacent strip, then continues down to the lower jaw.

Two nerves supply the ophthalmic dermatome and carry information anteriorly. Medially, the supratrochlear nerve begins anterior to the vertex. Laterally, the supraorbital nerve begins further posteriorly, ‘nearly as far back as the lambdoid suture’ (Davis and Davis, 1962)—commencing in proximity to fibers of the auriculotemporal nerve.

The supratrochlear and supraorbital nerves enter the orbit and form the frontal nerve which joins other branches to form the ophthalmic nerve which enters the skull through the superior orbital fissure. It joins the trigeminal ganglion, and the trigeminal nerve enters the pons. Fibers from the ophthalmic nerve join the chief sensory nucleus and the spinal trigeminal nucleus. Fibers from both nuclei communicate with the facial nucleus on the same side—the spinal trigeminal nucleus also communicates with the facial nucleus on the other side.

The facial nerve exits the pons and takes a complex path, passing through the facial canal and exiting the skull at the stylomastoid foramen. It then passes through the parotid gland and divides into branches. The temporal branch innervates the upper half of the orbicularis oculi, and the zygomatic branch innervates the lower half of that muscle.

Communication between the trigeminal and facial nuclei in the pons has been mentioned—at least five other connections between these nerves are described (Bernardini, 2021; Cobo et al., 2017; May and Warren, 2021).

We believe these components are sufficient to support a supraorbital nerve–triggered blink reflex.

The auriculotemporal nerve (a sensory branch of the mandibular nerve) gathers information from the scalp above and posterior to the ear (and other regions). It branches and may be depolarized during RMT determinations.

It joins the main trunk of the mandibular nerve and enters the skull via the foramen ovale. The sensory fibers of the mandibular nerve enter the trigeminal ganglion, and as components of the trigeminal nerve, enter the lateral pons. They reach the chief sensory nucleus of the trigeminal nerve and project fibers to the motor nucleus of trigeminal nerve.

Motor fibers leave the pons and travel in the mandibular nerve, pass under the trigeminal ganglion, and leave the skull through the foramen ovale. The masseter nerve emerges, passes through the mandibular notch, and enters the deep surface and innervates the masseter muscle. We believe these components are sufficient to support an auriculotemporal nerve–triggered blink reflex.

## Discussion and conclusion

Clinical observation and the listed anatomical structures indicate the presence of two undescribed reflexes—an auriculotemporal nerve–triggered bite reflex and a supraorbital nerve–triggered blink reflex.

Two questions arise. First, do these movements satisfy the definition of a ‘reflex’? The answer is in the affirmative—a reflex is an action performed in response to a stimulus, without conscious thought.

Second, are these are distinct reflexes or are they mere imitations of the jaw jerk and the corneal reflexes? The jaw jerk reflex is triggered by sudden stretching of the masseter muscle—the afferent message being initially carried by the masseter nerve. What we have called the auriculotemporal nerve–triggered bite reflex is triggered by the electrical depolarization of another nerve. While both reflexes involve contraction of the masseter muscle, they are trigged differently and the afferent information is initially carried by different nerves.

The corneal reflex involves the blinking of both eyes in response to noxious stimulation of specialized receptors (Belmonte et al., 2004)—the information being carried back by the nasociliary branch of the ophthalmic branch of the trigeminal nerve. What we have called the supraorbital nerve–triggered blink reflex is triggered by electrical depolarization of axons of the supraorbital branch of the trigeminal nerve, which conveys the information back to the brain. Both reflexes involve contraction of the orbicularis oculi, but one is triggered by noxious stimuli and the other by non-noxious stimuli, and the pathways back to the brain are different.

Currently, when we are performing an RMT determination, and the patient displays biting or blinking, we are inclined to think we are doing something wrong and have a sense we should apologize. With this new understanding, we would be better advised to simply inform the patient a reflex is being unavoidably triggered, and while this might be a little disconcerting, there is no danger of a serious outcome.

A related question: do the mechanisms described have relevance to the blinking and biting sometimes observed with stimulation of the dorsolateral prefrontal cortex (DLPFC). The supraorbital nerve passes directly over the DLPFC, thus the above explains any blinking when this area is stimulated. With respect to biting action, the auriculotemporal nerve reaches close to the DLPFC, but the zygomaticotemporal nerve (a branch of the maxillary nerve—second branch of the trigeminal) may be more important. Depending on individual variation, both have the potential to reflexively activate the masseter (biting) muscle.
